# Establishment and validation of a nomogram for progression to diabetic foot ulcers in elderly diabetic patients

**DOI:** 10.3389/fendo.2023.1107830

**Published:** 2023-04-04

**Authors:** Zhuce Shao, Zilong Wang, Shuxiong Bi, Jianguo Zhang

**Affiliations:** ^1^Third Hospital of Shanxi Medical University, Shanxi Bethune Hospital, Shanxi Academy of Medical Sciences, Tongji Shanxi Hospital, Taiyuan, China; ^2^Department of Orthopedics, Shanxi Bethune Hospital, Shanxi Academy of Medical Sciences, Tongji Shanxi Hospital, Third Hospital of Shanxi Medical University, Taiyuan, China; ^3^Department of Orthopedics, Tongji Hospital, Tongji Medical College, Huazhong University of Science and Technology, Wuhan, China

**Keywords:** diabetic foot, elderly, diabetes, nomogram, prediction

## Abstract

**Background:**

Many diabetic patients develop and progress to diabetic foot ulcers, which seriously affect health and quality of life and cause great economic and psychological stress, especially in elderly diabetic patients who often have various underlying diseases, and the consequences of their progression to diabetic foot ulcers are more serious and seriously affect elderly patients in surgery. Therefore, it is particularly important to analyze the influencing factors related to the progression of elderly diabetic patients to diabetic foot, and the column line graph prediction model is drawn based on regression analysis to derive the influencing factors of the progression of elderly diabetic patients to diabetic foot, and the total score derived from the combination of various influencing factors can visually calculate the probability of the progression of elderly diabetic patients to diabetic foot.

**Objective:**

The influencing factors of progression deterioration to diabetic foot in elderly diabetic patients based on LASSO regression analysis and logistics regression analysis, and the column line graph prediction model was established by statistically significant risk factors.

**Methods:**

The clinical data of elderly diabetic patients aged 60 years or older in the orthopedic ward and endocrine ward of the Third Hospital of Shanxi Medical University from 2015-01-01 to 2021-12-31 were retrospectively analyzed and divided into a modeling population (211) and an internal validation population (88) according to the random assignment principle. Firstly, LASSO regression analysis was performed based on the modeling population to screen out the independent influencing factors for progression to diabetic foot in elderly diabetic patients; Logistics univariate and multifactor regressions were performed by the screened influencing factors, and then column line graph prediction models for progression to diabetic foot in elderly diabetic patients were made by these influencing factors, using ROC (subject working characteristic curve) and AUC (their area under the curve), C-index validation, and calibration curve to initially evaluate the model discrimination and calibration. Model validation was performed by the internal validation set, and the ROC curve, C-index and calibration curve were used to further evaluate the column line graph model performance. Finally, using DCA (decision curve analysis), we observed whether the model could be used better in clinical settings.

**Results and conclusions:**

(1) LASSO (Least absolute shrinkage and selection operator) regression analysis yielded a more significant significance on risk factors for progression to diabetic foot in elderly diabetic patients, such as age, presence of peripheral neuropathy, history of smoking, duration of disease, serum lactate dehydrogenase, and high-density cholesterol; (2) Based on the influencing factors and existing theories, a column line graph prediction model for progression to diabetic foot in elderly diabetic patients was constructed. The working characteristic curves of subjects in the training group and their area under the curve (area under the curve = 0.840) were also analyzed simultaneously with the working characteristic curves of subjects in the external validation population and their area under the curve (area under the curve = 0.934), which finally showed that the model was effective in predicting column line graphs; (iii) the C-index in the modeled cohort was 0.840 (95%CI: 0.779-0.901) and the C-index in the validation cohort was 0.934 (95%CI: 0.887-0.981), indicating that the model had good predictive accuracy; the calibration curve fit was good; (iv) the results of the decision curve analysis showed that the model would have good results in clinical use; (v) it indicated that the established predictive model for predicting progression to diabetic foot in elderly diabetic patients had good test efficacy and helped clinically screen the possibility of progression to diabetic foot in elderly diabetic patients and give personalized interventions to different patients in time.

## 1 Introduction

Diabetes mellitus is a common complication associated with disorders of glucose and insulin metabolism (1). Diabetes mellitus is a series of metabolic disorders characterized by hyperglycemia (2). As a very common chronic disease, diabetes is of increasing concern to patients and physicians worldwide. Some studies show that currently, 3.882 billion people have diabetes, and the total number in the world is increasing (3). Public health economic pressures from diabetes are also increasing, and the cause of these is more likely to come from complications of diabetes, with diabetics at higher risk of microvascular complications such as nephropathy, neuropathy and retinopathy. Complicated cardiovascular diseases such as ischemic heart disease, stroke and peripheral arterial disease. One study found that nearly 20% of diabetic patients will eventually develop a diabetic foot during their lifetime. The diabetic foot, in turn, can bring further more serious consequences and complications (4). More urgently, many elderly patients have many underlying diseases themselves, such as hypertension, diabetes and cerebral infarction, etc. Elderly patients with concomitant diabetes should receive more attention and more and better preventive measures for the complications brought about by diabetes. Among them, the consequences of developing diabetic foot in elderly diabetic patients are more serious and require more urgent treatment or timely prevention. Foot complications are among the most serious and costly complications of DM, eventually leading to amputation of the lower extremity or part of it due to foot ulcers (5). The diabetic foot is one of the most serious complications of diabetes and is defined as a group of syndromes of neuropathy, ischemia and infection leading to tissue breakdown and possible amputation. If a foot ulcer is untreated and fails to heal, it can become infected and 5-24% of foot ulcers will result in limb amputation within 6 to 18 months of the first evaluation (6, 7). More than half of the non-traumatic amputations are due to diabetic foot, which shows that the harm and economic pressure caused by diabetic foot is great, and the persecution of diabetic patients should not be ignored, and the subsequent family and psychological impact should not be underestimated as well. Moreover, the life expectancy of elderly diabetic foot patients is further affected by amputation, and the mortality rate is greatly increased.

Although a number of scholars have conducted studies on the progression of diabetes and diabetic foot, the mechanisms and risk factors of how elderly diabetic patients progress to diabetic foot step by step have not been conclusively established, and few studies have been conducted on the development of diabetic foot in this group of elderly patients. One study, a national survey in the United States, showed that smoking was more common in white Americans and American Indians than in other racial groups such as blacks and Asians (8). Previous studies have identified smoking as a risk factor for diabetic foot ulcers because daily tissue hypoxia may lead to vascular and neuropathic disease in the lower extremities of diabetic patients (9). A number of studies have concluded that there are differences in age, duration of diabetes, BMI distribution, hypertension, and diabetic retinopathy between patients with and without diabetic foot ulcers, and these findings suggest some of the potential influencing factors for the development of diabetic foot in patients with diabetes. The magnitude of the effect of a patient’s obesity on the risk of developing ulcers in the diabetic foot is inconclusive. Given previous studies suggesting that obesity may be associated with diabetic foot ulcers (10, 11). Some prospective studies have concluded that there is no significant relationship between BMI and the development of diabetic foot (12), There are also findings showing that patients with BMI <25 kg/m2 and BMI ≥45 kg/m2 are associated with a higher risk of developing diabetic foot ulcers (13). Studies have shown that impaired microcirculation in diabetic patients may lead to dysfunctional vasodilation leading to secondary complications in the lower extremities, and diabetic foot patients with retinopathy have higher levels of diabetic biomarkers (14–16), These results suggest an association between retinopathy and diabetic foot ulcers. In addition, some studies have found that men with diabetes are more likely to develop diabetic foot than women with diabetes, and this gender variability has been suggested to be related to more labor and physical activity in men (17, 18).

## 2 Objects and methods

### 2.1 Patients

299 diabetic patients, aged 60 years or older, were selected from January 2015 to December 2021 in the Department of Orthopedics and Endocrinology, Third Hospital of Shanxi Medical University. Retrospective analysis of 299 patients hospitalized for diabetes mellitus or diabetic foot from January 2015 to December 2021. These 299 patients were randomly divided internally into a modeling population and a validation population. We randomly collected a total of 299 elderly diabetic patients, of which 211 patients randomly collected at the beginning were the modeling population and 88 patients randomly collected later were the validation population according to time allocation. In the modeling population, there were 102 male patients and 109 female patients; there were 53 patients with diabetic foot and 158 patients with non-diabetic foot. The internal validation population consisted of 88 patients, of whom 28 were diabetic foot patients, while a total of 60 were non-swollen patients, and other specific information can be found in **Table 1**.

**Table 1:** Patient demographic characteristics of the modeled population.

Features	Modeling population (n=211)/n (%)	Validation population (n=88)/n (%)	P value
Age (%)			0.266
60-65	92 (43.6)	48(54.5)	
66-75	77 (36.5)	29(33.0)	
>75	42 (19.9)	11(12.5)	
Sex (%)			0.533
Male	102 (48.3)	42(47.7)	
Female	109 (51.7)	46(52.3)	
Duration of Diabetes/year (%)			0.455
<5	55 (26.1)	25(28.4)	
5~10	74 (35.0)	33(37.5)	
>10	82 (38.9)	30(34.1)	
Alcohol (%)			0.166
YES	19 (9.0)	82(93.2)	
NO	192 (91.0)	6(6.8)	
Triglycerides (%)			0.312
0~1.7	163 (77.3)	65(73.9)	
>1.7	48 (22.7)	23(26.1)	
BMI (%)			0.123
<18.5	23 (10.9)	11(12.5)	
18.5-23.9	165 (78.2)	60(68.2)	
>24.0	23 (10.9)	17(19.3)	
Lactate dehydrogenase (%)			0.335
≤245	185 (87.7)	76(86.4)	
>245	26 (12.3)	12(13.6)	
Peripheral neuropathy (%)			0.563
YES	47 (22.3)	62(70.5)	
NO	164(77.7)	26(29.5)	
Smoking (%)			0.566
YES	160 (75.8)	17(19.3)	
NO	51 (24.2)	71(80.7)	
Total cholesterol (%)			0.344
≤6.0mmoI/L	161 (76.3)	21(23.9)	
>6.0mmoI/L	50(23.7)	67(76.1)	
High-density cholesterol (%)			
≤2.0mmol/L	123 (58.3)	50(56.8)	
>2.0mmol/L	88 (41.7)	38(43.2)	
High blood pressure (%)			0.243
NO	129 (61.1)	58(65.9)	
YES	82 (38.9)	30(34.1)	
Diabetic foot (%)			0.169
NO	158 (74.9)	60(68.2)	
YES	53 (25.1)	28(31.8)	

Both the modeling and validation populations passed informed consent.

The study of this columnar map prediction model was reviewed and approved by the ethics committee of the Third Affiliated Hospital of Shanxi Medical University.

The following diagnostic criteria were selected to meet: the criteria for diabetic foot established by the World Health Organization for retrospective analysis of the occurrence of DF, and relevant clinical data were collected for the analysis.

The inclusion criteria were as follows.

1. meeting the diagnostic criteria for diabetes mellitus established by the World Health Organization. 2. patients with complete case data and clinical examination data, and those who gave informed consent and voluntarily participated in the survey.

Exclusion criteria were as follows.

1. patients with malignancy, myocardial infarction or other serious infectious diseases and cognitive dysfunction. 2. those without clear diagnostic findings

### 2.2 Methods

Information about patients in the modeling and validation populations was collected by reviewing electronic medical records.

General information: including age, gender, height, BMI, etc.; ② Clinical information: duration of disease, presence of hypertension, etc.

#### 2.2.1 Main observation indicators

(i) the results of univariate and multifactorial logistics regression analysis of the development of diabetic foot in elderly diabetic patients; (ii) the construction of the column line graph prediction model; (iii) the evaluation results of the column line graph prediction model; (iv) the internal validation of the column line graph prediction model.

#### 2.2.2 Statistical analysis

LASSO (Least absolute shrinkage and selection operator) regression analysis was first performed using the (glmnet) package of R language software (version 4.0.5) to screen out statistically significant variables, i.e., age, presence of peripheral neuropathy, history of smoking, duration of disease, serum lactate Dehydrogenase, high-density cholesterol and other 10 variable factors were influential risk factors for progression of diabetes to diabetic foot ulcers in the elderly. A multifactorial logistics regression analysis was then performed using SPSS (version 25.0). Further, column line graph prediction models were drawn using the six screened risk factors by R language software “car”, “rms”. In order to evaluate the accuracy of the developed prediction models, the models were also evaluated simultaneously by producing C-index, calibration curves and using receiver operating characteristic (ROC) curves by R language software. Finally, in order to make the established model can be better used in clinical work, it is validated with DCA (Decision curve analysis) decision curve to determine whether the model has better performance in clinical work. In this case, the calibration curve has a 45° diagonal line, and the closer the other line produced is to this diagonal line, the better the model is. In addition, the ROC curve is calculated mainly by its AUC area, and different ranges of AUC area represent different meanings, where the model has no predictive power: AUC < 0.50; moderate accuracy: 0.50 < AUC ≤ 0.70; moderate to high accuracy: 0.70 < AUC ≤ 0.90; high accuracy: > 0.90.

## 3 Results

### 3.1 Analysis of the number of participants The medical records of the 211 modeled populations were analyzed

The number of participants and groups included in the observation, the number of participants and groups entered into the outcome analysis, and whether there was any shedding, if so please explain why.

**Figure 1:**
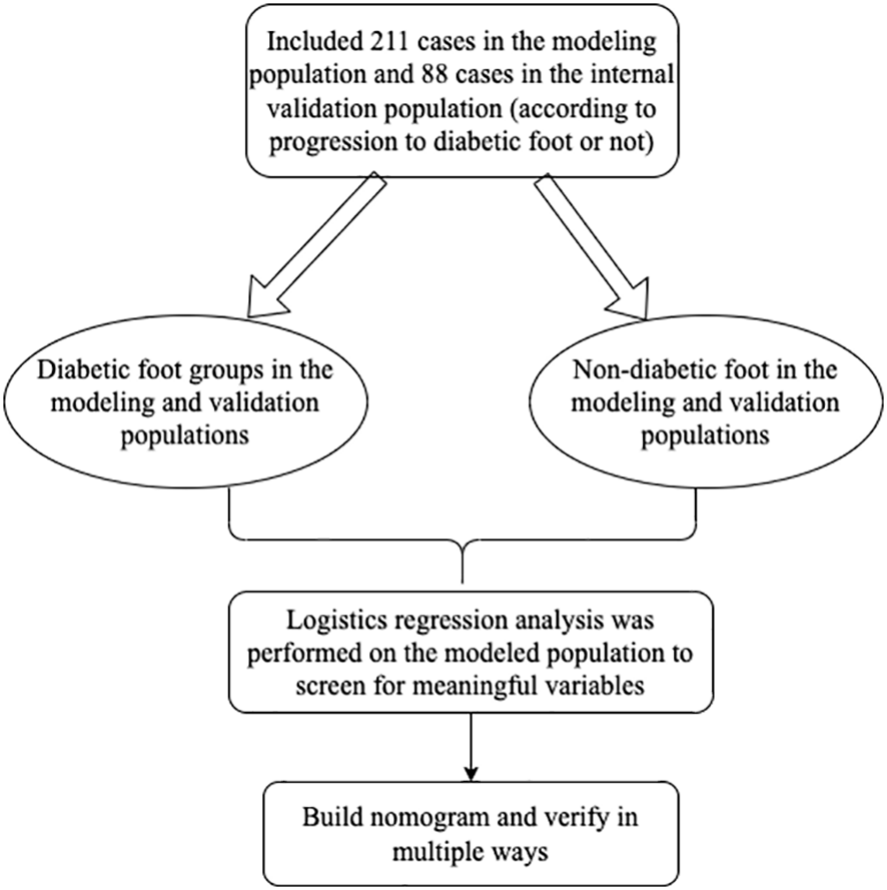
Flow chart.

The flow chart of the trial can be seen in **Figure 1**.

### 3.2 Baseline information

We found that 48.3% of the patients in the modeled population were male and 51.7% were female. The majority of patients (78.2%) had a BMI between 18.5 and 23.9, and 38.9% had a disease duration of more than 10 years, while this data for the validation population showed that 34.1% of patients had a disease duration of more than 10 years. Specific information for all modeled and validated populations is shown in **Table 1**. And there was no significant comparison of baseline information between the two groups.

### 3.3 Screening of risk factors for progression to diabetic foot ulcers in elderly diabetic patients

**Figure 2:**
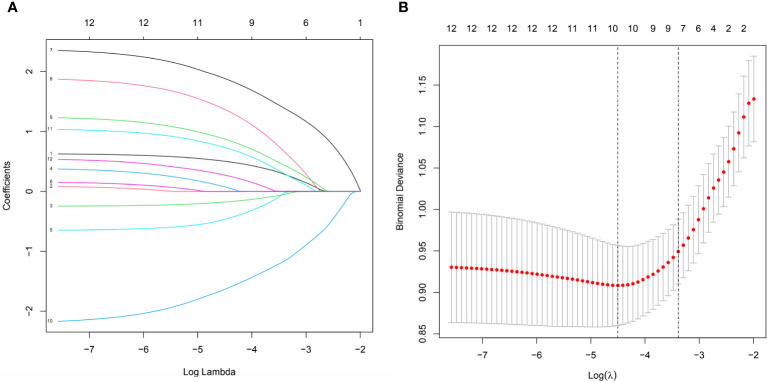
**(A)** All parameters were included in the LASSO analysis and coefficient curves were plotted. **(B)** Binomial deviations were plotted using the LASSO binary logistic regression model, which ultimately showed that 10 parameters were significant.

The clinical data collected from elderly diabetic patients (diabetic foot and non-diabetic foot) were subjected to LASSO regression analysis, and 10 factors were found to be important influences on the progression to diabetic foot ulcers in elderly diabetic patients, including age, presence of peripheral neuropathy, smoking or not, high-density cholesterol, lactate dehydrogenase, total serum cholesterol, history of alcohol consumption, age, presence of hypertension, and triglycerides factors, as shown in **Figure 2** of the LASSO regression analysis.

### 3.4 Results of logistics regression analysis of progression to diabetic foot ulcers in elderly diabetic patients

**Table 2** shows the results of the univariate and multifactorial logistics regression for the modeled population, which reveals statistically significant effects of age, presence of peripheral neuropathy, presence of smoking, high-density cholesterol, lactate dehydrogenase, and total serum cholesterol. The details can be seen in **Table 2**.

**Table 2:** Logistics regression univariate and multifactor analysis.

Variables	Univariate-analysis	P value	Multivariate-analysis	P value
	OR (95%CI)		OR (95%CI)	
Age	1.894 (1.219- 3.045)	0.006	1.882 (<0.001-3.352)	0.025
sex	1.149 (0.616-2.148)	0.661	1.099 (<0.001-2.508)	0.823
Duration of diabetes	0.685 (0.451-1.021)	0.07	0.780 (<0.001-1.260)	0.314
alcohol	1.878 (0.594-8.315)	0.333	1.484 (<0.001-8.361)	0.612
Triglycerides	0.669 (0.332-1.386)	0.267	0.519 (<0.734-1.311)	0.164
BMI	0.637 (0.414-0.990)	0.04	1.179 (<0.001-2.174)	0.582
dehydrogenase	6.400 (2.725-15.725)	<0.001	10.855 (<0.001-38.268)	<0.001
Peripheral neuropathy	2.228 (0.981-5.751)	0.07	6.702 (<0.001-23.160)	0.001
Smoking	3.943 (1.603-11.905)	0.006	3.506 (<0.001-12.677)	0.035
Total cholesterol	1.099 (0.788-1.531)	0.001	0.110 (<0.001-0.450)	0.007
High-density cholesterol	2.454 (1.260-5.009)	0.01	2.867 (<0.001- 6.947)	0.016
High blood pressure	1.324 (0.697-2.579)	0.398	1.739 (<0.001-4.304)	0.219

### 3.5 Establishment of a nomogram to predict the risk of progression of diabetic foot ulcers in elderly diabetic patients

**Figure 3:**
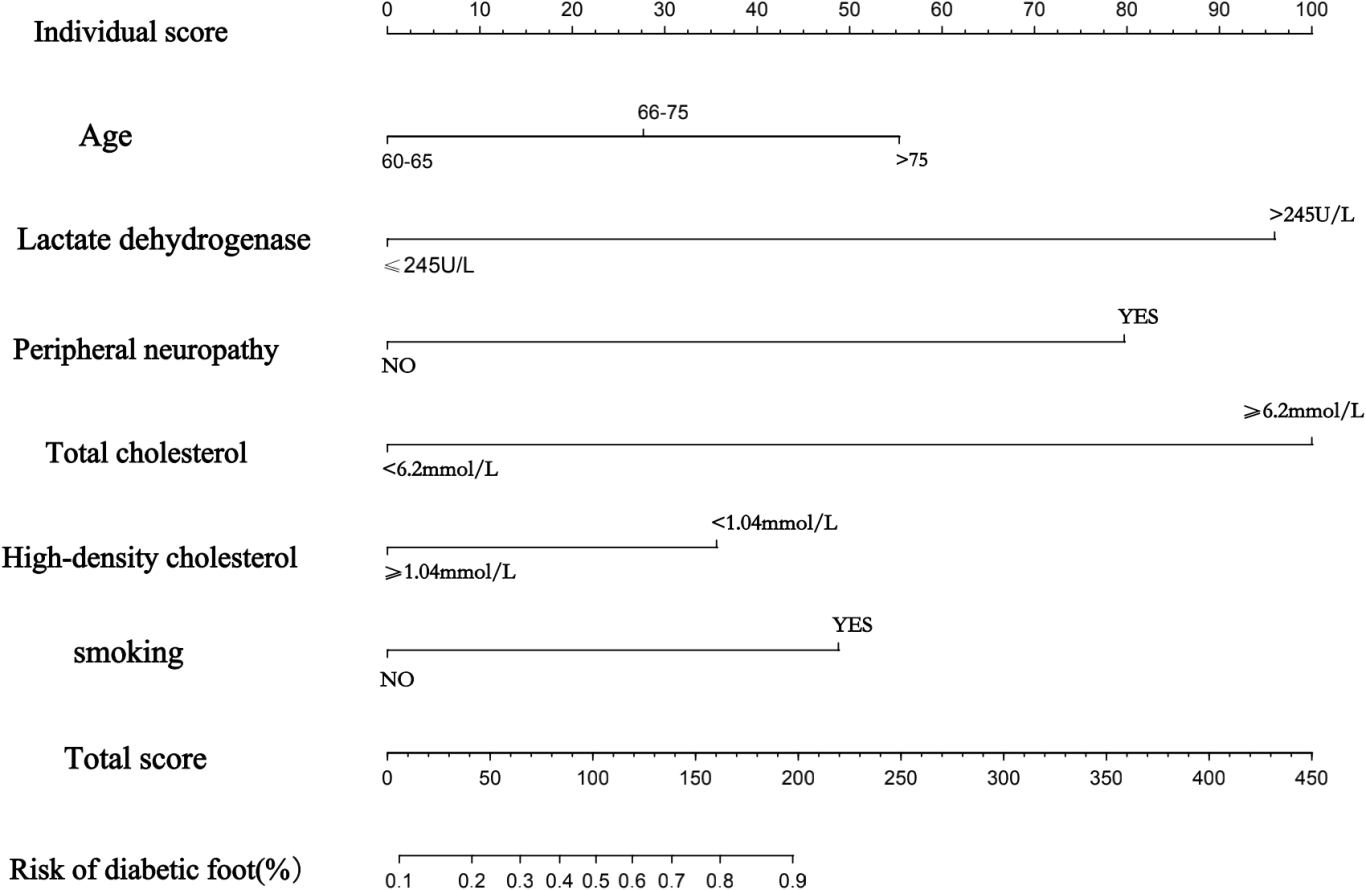
Nomogram drawn with 6 variables.

Ultimately, a nomogram was drawn based on the six statistically significant risk factors of age, presence of peripheral neuropathy, presence of smoking, high-density cholesterol, lactate dehydrogenase, and total serum cholesterol of the patients (**Figure 3**). **Figure 3** predicts the probability of progression to diabetic foot ulcers in older diabetic patients. In the nomogram we created, the effect of each variable on the endpoint event is reflected in the respective row length and the corresponding score. Individualized scores are available for different patients. The total score associated with each variable constitutes the probability of progression to a diabetic foot ulcer in older diabetic patients. We found that of all the factors included, the patient’s total cholesterol, presence of peripheral neuropathy, and lactate dehydrogenase had a more significant effect on progression to diabetic foot ulcers in elderly diabetic patients, followed closely by age and whether or not they smoked also contributed significantly to the development of swelling. Different patients have different individualized scores, giving clinicians more over treatment decisions or better interventions to take.

### 3.6 ROC curve to assess the accuracy of the prediction model

**Figure 4:**
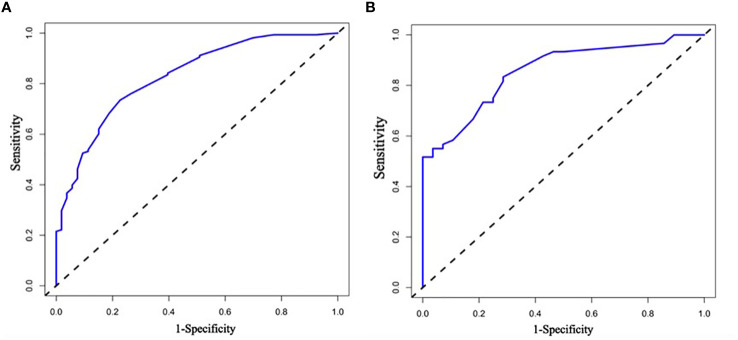
**(A, B)** Are the ROC curves of nomogram of modeling group and verification group respectively. ROC curve is the subject work characteristic curve; AUC is the effective area under the curve. the calculation of ROC curve is mainly expressed by its AUC area, and different ranges of AUC area represent different meanings.

Receiver Operating Characteristic (ROC) curves were used to evaluate the prediction performance of the column line graphs, and the ROC curve and its AUC area (AUC=0.840) for the training group were calculated by R language software, and the ROC curve and its AUC (AUC=0.934) for the validation group were also analyzed at the same time, which finally showed that the model was predictive ability of the line graph was effective. **Figures 4A, B** show the ROC curves of the modeling group and the validation group nomogram, respectively.

### 3.7 Graphical calibration method and C-index to validate the predictive ability of the column line graph model

**Figure 5:**
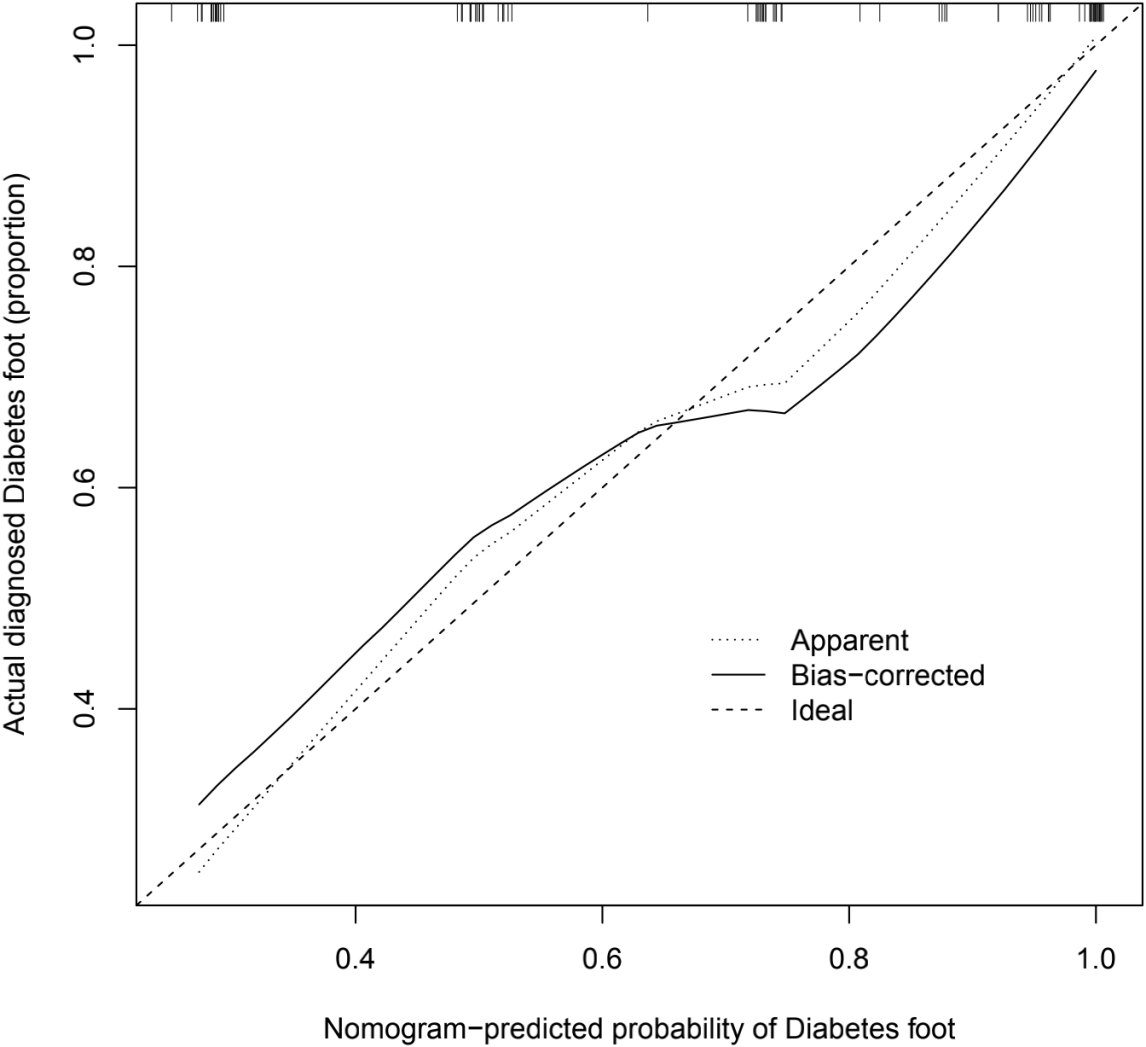
Calibration diagram. The calibration graph is a way to assess the accuracy of the prediction model for the column line graph. The significance of the calibration chart is that the closer the actual line in the chart is to the 45 degree diagonal the more accurate the prediction model is, and this image reflects the better accuracy of the prediction model we have built.

The calibration graphs of the training and validation groups (**Figure 5**) were also used to assess the accuracy of the prediction results of the column line graphs relative to the actual occurrence. Ideally, the calibration curve is a diagonal line; at this point, the predicted probability is equal to the true probability. The calibration curve confirms the good agreement between the actual and predicted values (**Figure 5**). **Figure 5** shows the calibration curve for the column line graph; the blocks are close to the 45 degree line, indicating that the survival nomogram is well calibrated in the training set. In addition, we also calculated a C-index of 0.840 (95%CI:0.779-0.901) in the training modeling cohort and 0.934 (95%CI:0.887-0.981) in the validation group, indicating that the prediction accuracy of the model was quite good.

### 3.8 Decision curve DCA validation for clinical applicability

**Figure 6:**
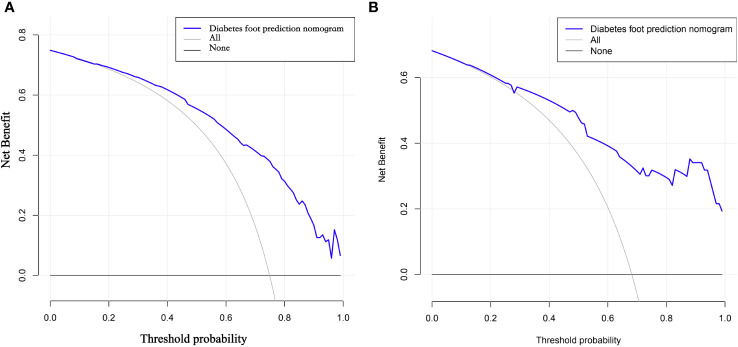
**(A, B)** DCA curves of the modeling group and validation group nomogram. DCA curves refer to decision curve analysis, which allows assessing the efficacy of the model for clinical use. The decision curve shows that if the probability of swelling occurs between 29% and 70%, the net benefit level of applying the columnar line graph is significantly higher than other options with better clinical benefit. Therefore, the DCA curves we plotted indicate that the predictive model of column line graphs derived from the modeled population is clinically useful.

The DCA curves show that the predictive models derived from the modeled population for the column line plots are clinically useful (**Figure 6**).

## 4 Discussion

Older patients with diabetic foot should receive more attention because they are always accompanied by other diseases and also, some studies have found that older diabetic patients are at higher risk of developing diabetic foot ulcers and their incidence may be higher (19). The prevalence of diabetic foot is not low, roughly 6% (17). And many studies have been conducted to analyze the risk factors for the progression of diabetes to diabetic foot ulcers, and these studies point out that the development of diabetic foot ulcers is associated with Hb1AC, foot trauma, obesity and overweight, smoking or not, duration of diabetes, and increasing age (20–24).

This study established novel, convenient, and highly accurate columnar line graphs for predicting the probability of progression to diabetic foot ulcers in older adults with diabetes, and to our knowledge, no study has ever analyzed and produced a columnar line graph prediction model on predicting progression to diabetic foot ulcers in older adults with diabetes. Columnar line plots also showed satisfactory agreement in both the modeled and validated populations, suggesting good clinical applicability. Columnar line plots are widely used for the prediction of various diseases or complications, mainly because of their ability to reduce statistical prediction models to estimates of simple numbers of event probabilities (e.g., diagnosis or recurrence) tailored to the profile of individual patients. It can inform clinical decision making (25). Column line graphs meet the need for integrated biological and clinical models and the quest for personalized medicine that can provide individualized predictions for individual patients (26).

From the results of our study, age, presence of peripheral neuropathy, smoking or not, high-density cholesterol, lactate dehydrogenase, and total serum cholesterol were the most relevant variable factors affecting the progression of diabetes to diabetic foot ulcers in older patients with diabetes. This also has similarities with some of the previous studies done above. First, the age of diabetes in elderly patients can influence the development of diabetic foot, probably because the longer the duration of the disease, the greater the accumulation of long-term progression of diabetes and the chance of complications, which can be verified from some later studies, which also suggests that clinicians should start to intervene or treat the progression of diabetes at an early stage when guiding patients, as the duration of diabetes and patient’s age, it may bring about drawbacks similar to diabetic foot ulcers and the like. Then, clinicians do not recommend that patients delay treatment while ensuring that they receive the best possible treatment. Then, the effect of whether or not to smoke on the progression of diabetes to diabetic foot is also supported by many studies on the effect of smoking on diabetic microangiopathy, where smokers have diminished vasodilatation of different stimuli (mainly endothelium-dependent) to the skin microvasculature, further reducing the already reduced blood flow in the diabetic microcirculation, which in turn leads to impaired vascular activity eventually progressing or exacerbating diabetic foot ulcers, (27–29) Smoking also leads to the production of reactive oxidants in leukocytes, leading to a local inflammatory response that affects the development of the diabetic foot (30, 31). And our study, further proves that smoking is also an important risk factor for the development of diabetic foot ulcers in older diabetic patients, and even we believe that smoking has a greater impact on the progression to diabetic foot in older diabetic patients than in younger diabetic patients, because the microvasculature is more fragile and more prone to problems in older patients, so older diabetic patients who have a history of chronic and persistent smoking are only more susceptible to complications of diabetes. Peripheral neuropathy in elderly diabetic patients has also been shown to be one of the important influencing factors in the progression to diabetic foot through our study, which also found in numerous previous studies that peripheral neuropathy accounts for about 30% of diabetic patients and even more than half of type 2 diabetic patients over 60 years of age (32, 33). Nearly 80% of patients with diabetic foot ulcers have peripheral neuropathy (34). Oxidative stress is considered to be the ultimate mechanism of cellular damage in diabetic neuropathy, which is characterized by high levels of sustained generation of reactive oxides (ROS) and ultimately damages the nervous system leading to diabetic foot (35, 36). Similar to our findings, the close correlation between peripheral neuropathy and diabetic foot ulcers has also been demonstrated (35, 37). Moreover, our study further demonstrates that peripheral neuropathy is also a risk factor for progression to diabetic foot in the older age group of diabetic patients and is closely associated with its development. Our study suggests that clinicians need to pay extra attention to peripheral neuropathy in older diabetic patients and intervene early to prevent further progression to diabetic foot ulcers in older diabetic patients. Finally, our study also found that high-density cholesterol, lactate dehydrogenase, and total serum cholesterol were all associated with progression in elderly diabetic patients, similar to our findings, and it has also been suggested that low levels of HDL contribute to the development of diabetic foot ulcers by increasing the risk of diabetic peripheral neuropathy (38). Whereas our findings suggest a correlation between lactate dehydrogenase and total serum cholesterol and the progression of diabetes mellitus to diabetic foot ulcers in the elderly, there are indeed fewer studies of this type, but a few scholars have also performed the effect of lactate dehydrogenase or total serum cholesterol on diabetes mellitus, and there are those who believe that diabetes mellitus and diabetic foot ulcers are essentially the result of disorders of blood glucose and lipids, so their relationship with the development of diabetic foot cannot be ruled out. The relationship between them and the development of diabetic foot cannot be ruled out (39, 40). This seems to require further findings and studies in later prospective clinical trials.

The analysis of the ROC curves and their AUC areas revealed that the predictive model of the column line graphs we have drawn has good accuracy. Moreover, we also used the C-index and calibration plots to again validate the good predictive accuracy of the column line plot for the progression of diabetes to diabetic foot ulcers in the elderly.

The limitation of this study is that a multicenter study was not conducted, and cooperation of multiple centers can be sought in subsequent studies to further improve the accuracy of the model.

For future studies, we hope that more scientific results may be obtained by prospectively investigating the progression of geriatric diabetes to diabetic foot ulcers.

## 5 Conclusion

We have developed and are validating a new nomogram for predicting the risk of developing diabetic foot ulcers in older diabetic patients. After our study, we found that Age, dehydrogenase, Peripheral neuropathy, Smoking, Total cholesterol, and High-density cholesterol are meaningful risk factors for the development of diabetic foot ulcers in elderly diabetic patients after multiple modalities. validation showed that the nomogram we established has good accuracy and precision and has good predictive value. This is particularly useful for those working with diabetic foot wounds and care, and our study could suggest some hypotheses about the occurrence of diabetic ulcers for clinicians to accept. It could provide a more effective protocol for intervening in the progression to diabetic foot ulcers in older diabetic patients.

## 6 Limitations

This study has limitations. This is a single center retrospective study that affects patients and produces selection bias. Finally, the cases in this study are small samples from the same hospital. We suggest that in the follow-up study, it is better to conduct a prospective study with a large sample from multiple centers.

## Data availability statement

The original contributions presented in the study are included in the article/supplementary material. Further inquiries can be directed to the corresponding author.

## Ethics statement

The studies involving human participants were reviewed and approved by Medical Ethics Committee of the Third Hospital of Shanxi Medical University. The patients/participants provided their written informed consent to participate in this study. Written informed consent was obtained from the individual(s) for the publication of any potentially identifiable images or data included in this article.

## Author contributions

ZS and JZ designed and conceived the study, ZS wrote this paper. ZW and SB revised the article. All authors contributed to the article and approved the submitted version.
